# On the Treatment of Typhoid Forms of Scarlatina by Tonics, Alcoholic Stimulants and Food

**Published:** 1860-09

**Authors:** Benjamin Cutter

**Affiliations:** Woburn


*On the Treatment of Typhoid Forms of Scarlatina by Tonics, Alcoholic Stimulants and Food.* Read before the Middlesex East District Medical Society, July 11th, 1860, and communicated for the Boston Medical and Surgical Journal. By Benjamin Cutter, M. D., of Woburn.

The severest types of scarlatina prevailed during the past win. ter in the town of Winchester, and the number of fatal cases was large for the sum of the affected. Several died very suddenly in two or three days after they were attacked by the disease, with very few if any of the premonitory symptoms of a mild or lighter character, as we generally see; stupor and coma, with difficult respiration, sometimes alternating with severe struggles for breath, and attempts to leave the bed, requiring the strength and efforts of two or three persons to keep the patient on the bed, and the clothes on the patient; great restlessness generally, and jactitation; dark hue skin, and enlargement of pupils; pulse excessively quick and weak.

Case I.—A child, of 7 years, was taken, Jan. 29th, with vomiting. Was seen in half an hour from attack of vomiting; had eaten breakfast. Found her with pulse 140; strawberry tongue; throat quite free from inflammation or swelling. Gave emetic of ipecac, to be followed by veratrum viride *pro re nata.* Emetic operated passably well, and the next day she was no worse. She continued without improvement another day, when typhoid symptoms developed themselves, and the case was decidedly getting worse. Tonics were administered, with food and stimulants; wine especially, which it was found difficult to administer on account of the increased swelling and soreness of the throat. When I found I could not give this by the mouth, I directed the mother to use the syringe. With a good deal of hesitation and much doubt, she injected a wineglass of Sicily Madeira wine, and as much thin starch, when the child was in a very restless condition, and opposed to the administration of the enema. The effect was very marked and happy—so much so, that, before morning, on a return of these sysmptoms, the mother repeated the enema on her own responsibility. At my next visit I found the patient rational, and the appearance in her favor very much improved. In a week the patient was convalescent.

Case II.—A younger child was attacked in the same manner, and treated much in the same way, with a happy result.

Case III.—A boy 8 years old, Irish, was severely seized, and died in five days. I gave an unfavorable prognosis from the very first, as the child was in a hot, crowded tenement, and could not have proper nursing or treatment.

Case IV.—Boy 11 years old. Was taken at school with vomiting. I found him with pulse 144, respiration 28, skin red, throat sore and swollen; breathing hard, with enlarged tonsils of former times. He took ipecac, was rubbed with lard, and sometimes sponged with water. The emetic did not operate very well, but he was more comfortable the next day. The day after, I found his symptoms all aggravated, and a strong typhoid character. I then put him on a course of stimulants, and as he could not swallow readily, and his stomach loathed everything, I had recourse to the syringe. His enemas, which were faithfully given, and readily tolerated, were of two fluid ounces of wine, with as much thin starch, or beef, mutton, and chicken tea, and were continued for a week or ten days, every four hours a part of the time; they were absorbed, perhpas digested, and very seldom moved the bowels· On one occasion he had 16 enemas before any come away. He had all the other appliances he seemed to require, and eventually made a slow recovery, retarded by a large abscess, that formed over the sterno-mastoid muscle, and was opened.

Case V.—A child. 3½ years old. Taken very severely, and died the second day I visited him, with marked cerebral symptoms.

Case VI.—A child, 6 years old. Severely seized; became relieved at the end of a week, when there was a return of canker in the throat, with renewed fever, and eventually she died, with pneumonic symptoms, and in the extremest state of emaciation. All means were used, and the stimulent practice fairly tried without success.

Case VII—An infant, nine months old, large of its age, was taken with scarlatina, without much rash or sore throat, but with great thirst, and vomiting. The disease seemed to expend its violence on the mucous membrane of the intestines, the symptoms appearing continuous with cholera morbus, and dysentery, causing a true gastro-enteritis. The little patient lived about four days, and water could not satisfy his thirst. He wanted the cup or spoon at his mouth constantly.

Case VIII.—An Irish child, one year old. I expected it would die from want of proper nursing. I tried to favor the prejudices of the mother, and prescribed wine. In a week I stopped my visits, as the child appeared convalescent. A fortnight after, I heard of the death of the child, but do not know whether any change took place in its symptoms.

Ten or twelve other cases, in Winchester, were treated chiefly with the stimulating hydro-carbons, mostly by the mouth, with success.

Case IX.— J. B., 17 years old. Was taken the 20th of May. Saw him first on the 22d. Throat red, shining, some stringy mucus therein; rash out fully; head aches; hot; has had pepper tea. Gave him ipecac to vomit, and the veratrum viride afterwards.

May 23d.—Used the chlorine mixture, ice-water and bladder to the head.

24th.—Purged him, and gave wine. Inclined to be crazy, and sitting up.

25th.—Much canker in the throat; no cloths on; picking at things. Ordered the wine and water, wine whey and brandy. Mother was unwilling to give stimulants sufficiently freely.

26th.—Found that the wine had been disused since last visit, as his mother thought he was worse for taking it, causiug more heat and fever. He was crazy, had subsultus, and was continually trying to get out of the bed, and once succeeded in getting partly out of the window. For two days he had a male nurse, who mined the mother in giving the medicines. I told her that her fears were needless, as I had never seen fever made higher in these cases by the wine, and I could not see why the wine had not benefited him more. I increased the dose, and that night he had three injections of half a pint of Sic. Madeira wine with beef tea. He slept, and in the morning was free from subsultus and delirium. His brother, who watched that night, advised me to increase the quantity of wine still more. Pulse was 100; respiration 28.

For another week he continued to improve, when most of his old symptoms returned, and with them the subsultus, and I am satisfied that this second attack was owing to the fears and practice of his mother. His legs swelled, sores from sloughs came on both heel-cords, and a return to the strong stimulating practice was absolutely necessary. While improving, the week previously, his appetite became good, which was unexpected, and was considered a very good symptom in his favor. It was necessary to recur to the use of the wine injections with beef tea and sol. sulph. quiniæ. The legs were poulticed, and after cleansing the ded portions, dressed with adhesive plaster, and bandaged from the toes to the knees. He began to improve slowly, and is now convalescent. During his sickness he has used three gallons of Sicily Madeira wine, besides brandy and gin. The brandy would be retained on the stomach better than anything else, when nausea prevailed, as it did most of the time. When he could drink, the wine was very unpalatable.—For his throat he had a sponge, with strong solution of nitrate of silver· He took a chlorinated solution of chlorate of potash. Sometimes he had mur. tr. ferri, and the milder preparation of iron, the sol. tart. ferri et potassæ.—*Boston Medical & Surgical Journal.*

